# Pioneering Dementia Risk Prediction in Kenya: Insights from the AD‐Detect Kenya Project

**DOI:** 10.1002/alz70857_100253

**Published:** 2025-12-24

**Authors:** Chinedu T Udeh‐Momoh, Karen Blackmon, The AD‐DETECT Kenya Program Investigators

**Affiliations:** ^1^ Brain and Mind Institute, Aga Khan University, Nairobi Province, Nairobi, Kenya; ^2^ Wake Forest University School of Medicine, Winston‐Salem, NC, USA; ^3^ Dartmouth Hitchcock Medical Center | Psychiatry Department, Lebanon, NH, USA; ^4^ Brain and Mind Institute, Aga Khan University, Nairobi, Kenya

## Abstract

**Introduction:**

The AD‐Detect Kenya project represents a groundbreaking effort to enhance dementia risk prediction and phenotyping in Kenyan older adults, addressing critical gaps and limitations in culturally tailored diagnostic and prevention strategies for Alzheimer's disease and related dementias (ADRD) in Africa. The project leverages advanced deep learning analysis of cognitive, clinical, and bio‐psychosocial markers to lay the groundwork for high‐impact dementia clinical trials; a key example being the Africa‐FINGERS program, the first dementia risk reduction trial in Africa.

**Methods:**

The AD‐Detect Kenya initiative, emebedded within the Davos Alzheimer's Collaborative Global Cohorts Program employs multimodal, deep phenotyping approaches, integrating culturally validated neuropsychological tools, digital data capture (speech, olfactory systems) advanced imaging for differential diagnosis of dementia (MRI and FDG‐PET), and fluid biomarkers (e.g., blood, urine and saliva) to differentiate healthy controls from individuals with mild cognitive impairment and dementia across socio‐economic strata.

**Results:**

Preliminary findings highlight the significant role of cardiometabolic and vascular risk factors, such as hypertension, diabetes, and lifestyle determinants, on cognitive outcomes. Notably, a stark underdiagnosis of these conditions underscores the urgent need for targeted interventions. Cultural phenotyping has further identified markers uniquely relevant to African populations, enriching the understanding of dementia risk. The project's comparative framework includes cross‐cohort analysis with diverse international cohorts through the ADDI platform, facilitating insights into shared and distinct dementia risk profiles for Black Africans in both African and diasporic contexts. These data are critical for designing contextually appropriate interventions and informing global dementia prevention efforts. Results from these pilot evlauations will be presented.

**Conclusion:**

As a precursor to the Africa‐FINGERS program, AD‐Detect Kenya exemplifies how precision prevention strategies can be developed and implemented in low‐resource settings. The study underscores the value of dementia readiness cohorts in facilitating effective recruitment for tailored clinical trials. It also offers a scalable model that can be adapted to similar efforts in underrepresented populations worldwide.

This work not only contributes to reducing health disparities but also sets a benchmark for equitable and inclusive dementia research worldwide.

